# Draft Genome Sequence of *Pyrobaculum* sp. Strain 3827-6, a Hyperthermophilic Crenarchaeon, Isolated from a Kamchatka Hot Spring

**DOI:** 10.1128/mra.01159-22

**Published:** 2023-01-04

**Authors:** Evgenii N. Frolov, Anastasiia I. Maltseva, Alexander G. Elcheninov, Ilya V. Kublanov

**Affiliations:** a Winogradsky Institute of Microbiology, Federal Research Center of Biotechnology, Russian Academy of Sciences, Moscow, Russia; Portland State University

## Abstract

The genome of *Pyrobaculum* sp. strain 3827-6, a facultative autotrophic hyperthermophilic archaeon isolated from a Kamchatka hot spring, was sequenced and analyzed. Genome analysis predicted the dicarboxylate/4-hydroxybutyrate cycle and a [NiFe]-hydrogenase, as well as the tricarboxylic acid cycle, altogether determining the possibility of both autotrophic and heterotrophic growth of this strain.

## ANNOUNCEMENT

Strain 3827-6 was isolated by means of a dilution-to-extinction technique from an enrichment culture obtained by incubation of the 0.2-g unprocessed geothermally heated soil (78°C; pH 6.0; E_h_, 295 mV; depth, 30 cm; sampled at the Oil Site, Uzon Caldera, Kronotsky Nature Reserve, Kamchatka, Russia; 54°30.023′N, E160°00.088′E; elevation, 654 m; in August 2019) in 5 mL of anaerobically prepared liquid modified Pfennig medium (1) (pH 6.0) for 5 days at 92°C. H_2_ (the H_2_/CO_2_ ratio in the headspace of the tubes was 4:1) and elemental sulfur (10 g · L^−1^) were used as the electron donor and acceptor, respectively, during isolation and all autotrophic growth experiments. The growth experiments were performed in duplicates, and the growth yield was controlled using phase-contrast light microscopy. Besides chemolithoautotrophic growth, the isolate was capable to grow heterotrophically on the medium (1) with 100% CO_2_ headspace, yeast extract, peptone or tryptone (1 g · L^−1^ each), and sulfur (10 g · L^−1^) or thiosulfate (2 g · L^−1^).

For genomic sequencing, the strain 3827-6 was cultured for 5 days at 92°C and pH 6.0 on the same medium with elemental sulfur (10 g · L^−1^) and H_2_/CO_2_ (4:1) in the headspace. Sequencing and the data analysis were performed according to manufacturer recommendations and default parameters unless otherwise stated. Genomic DNA of the strain was isolated using DNeasy PowerLyzer microbial kit (Qiagen). DNA libraries were prepared using HyperPlus kit (KapaBiosystems). Illumina NovaSeq 6000 paired-end sequencing (2 × 100 bp) resulted in 29,430,240 raw reads with an average length of 101 bp. The raw reads were filtered (by quality and length) with CLC Genomics Workbench v.10 (Qiagen). The genome was assembled using SPAdes v.3.15.4 (2) and Unicycler v.0.4.9 (3). Preliminary assembly was done using Unicycler followed by SPAdes with the following parameters: –isolate mode with –trusted-contigs option (the contigs obtained from Unicycler were used as the trusted contigs). Contigs with a length of ≤ 500 bp or with low coverage (<10×) were eliminated from the final assembly. Genome assembly statistics was checked with QUAST v.5.0.2 (4). Completeness and contamination levels were measured using CheckM v.1.2.1 (5) with an archaeal-specific marker set. Genome annotation was performed using NCBI Prokaryotic Genome Annotation Pipeline v.6.3 (6).

The final assembly of the strain 3827-6 genome comprises 13 contigs with a total length of 2,302,599 bp, an *N*_50_ value of 1,414,246 bp, and a G+C content of 57.5%. Estimated completeness and contamination of the assembly were 99.07% and 0%, respectively. In total, 2,703 genes were predicted during annotation, including 2,653 protein-coding genes and 50 RNA genes (3 rRNA genes and 47 tRNA genes).

BLASTn (v.2.13.0, nonredundant [nr]/nucleotide database [7]) showed Pyrobaculum ferrireducens 1860^T^ is the nearest cultivated relative of strain 3827-6 with 99.60% 16S rRNA gene sequence identity. Unlike strain 3827-6, *P. ferrireducens* 1860^T^ was unable to grow chemolithoautotrophically (8) despite that both genomes contained all the genes encoding the dicarboxylate/4-hydroxybutyrate cycle. However, 1860^T^ lacked the genes encoding [NiFe]-hydrogenase and hydrogenase maturation factors, which seems to determine the inability of this strain to grow chemolithoautotrophically with hydrogen. The tricarboxylic acid cycle is responsible for the heterotrophic growth of strain 3827-6 on peptides.

### Data availability.

The whole-genome sequence was deposited in DDBJ/ENA/GenBank under the accession number JAOTQN000000000. The BioProject, BioSample, and SRA accession numbers are PRJNA886702, SAMN31139133, and SRR21876913, respectively.

## References

[1] Frolov EN, Zayulina KS, Kopitsyn DS, Kublanov IV, Bonch-Osmolovskaya EA, Chernyh NA. 2018. *Desulfothermobacter acidiphilus* gen. nov., sp. nov., a thermoacidophilic sulfate-reducing bacterium isolated from a terrestrial hot spring. Int J Syst Evol Microbiol 68:871–875. doi:10.1099/ijsem.0.002599. 29458537

[2] Bankevich A, Nurk S, Antipov D, Gurevich AA, Dvorkin M, Kulikov AS, Lesin VM, Nikolenko SI, Pham S, Prjibelski AD, Pyshkin AV, Sirotkin AV, Vyahhi N, Tesler G, Alekseyev MA, Pevzner PA. 2012. SPAdes: a new genome assembly algorithm and its applications to single-cell sequencing. J Comput Biol 19:455–477. doi:10.1089/cmb.2012.0021. 22506599 PMC3342519

[3] Wick RR, Judd LM, Gorrie CL, Holt KE. 2017. Unicycler: resolving bacterial genome assemblies from short and long sequencing reads. PLoS Comput Biol 13:e1005595. doi:10.1371/journal.pcbi.1005595. 28594827 PMC5481147

[4] Gurevich A, Saveliev V, Vyahhi N, Tesler G. 2013. QUAST: quality assessment tool for genome assemblies. Bioinformatics 29:1072–1075. doi:10.1093/bioinformatics/btt086. 23422339 PMC3624806

[5] Parks DH, Imelfort M, Skennerton CT, Hugenholtz P, Tyson GW. 2015. CheckM: assessing the quality of microbial genomes recovered from isolates, single cells, and metagenomes. Genome Res 25:1043–1055. doi:10.1101/gr.186072.114. 25977477 PMC4484387

[6] Tatusova T, DiCuccio M, Badretdin A, Chetvernin V, Nawrocki EP, Zaslavsky L, Lomsadze A, Pruitt KD, Borodovsky M, Ostell J. 2016. NCBI Prokaryotic Genome Annotation Pipeline. Nucleic Acids Res 44:6614–6624. doi:10.1093/nar/gkw569. 27342282 PMC5001611

[7] Altschul SF, Gish W, Miller W, Myers EW, Lipman DJ. 1990. Basic local alignment search tool. J Mol Biol 215:403–410. doi:10.1016/S0022-2836(05)80360-2. 2231712

[8] Slobodkina GB, Lebedinsky AV, Chernyh NA, Bonch-Osmolovskaya EA, Slobodkin AI. 2015. *Pyrobaculum ferrireducens* sp. nov., a hyperthermophilic Fe(III)-, selenate- and arsenate-reducing crenarchaeon isolated from a hot spring. Int J Syst Evol Microbiol 65:851–856. doi:10.1099/ijs.0.000027. 25510975

